# Post‐COVID Cognitive impairment in a large hospital‐based multi‐ethnic sample during the COVID‐19 pandemic

**DOI:** 10.1002/alz.091352

**Published:** 2025-01-09

**Authors:** Reem M. Neal, zhiyi Yang, Ihab Hajjar

**Affiliations:** ^1^ UT Southwestern Medical Center, Dallas, TX USA; ^2^ Emory University, Atlanta, GA USA

## Abstract

**Background:**

COVID‐19 has been associated with developing cognitive impairment. Individuals with cognitive impairment have a higher likelihood of progressing into dementia. Furthermore, studies have suggested that COVID‐19 is associated with an increased risk of developing Alzheimer’s disease (HR 2.03). Despite the increasing number of individuals developing post‐COVID cognitive impairment, the risk factors remain unclear.

**Method:**

We used the electronic health record data on 32,018 patients with at least one COVID‐19 infection from 2020‐2022. We defined cognitive events based on ICD 10 for memory loss, mild cognitive impairment, dementia, or any neurocognitive disorders. We looked at the temporal relation between these events and the COVID‐19 events and classified patients as either with or without cognitive impairment and whether it was an incident (i.e. only after COVID without any pre‐COVID cognitive impairment or pre‐existing cognitive impairment). We then explored potential correlates with COVID‐related cognitive impairments. We also evaluated the effect of race and sex on the possible risk factors for developing post‐COVID cognitive impairment. We used Multivariable logistic regression to determine the significance of associations.

**Result:**

In this study of 32018 patients with a COVID infection, and a mean age of 60, we found that the prevalence and incidence of post‐COVID cognitive impairment were higher in African American patients vs. White patients (prevalence 25% vs 23% incidence: 15% vs 13%) and male vs. female 39% vs. 33% patients. We also found a greater risk for post‐COVID cognitive impairment to be associated with delirium, recurrent COVID infection, kidney disease, and cardiovascular disorders, OR increased by 4.7, 2.4, 2.1 & 2.3 respectively (all *P<0.0001*). In contrast, we found that obesity, Remdesivir use, and prevalent psychiatric, and respiratory disorders decreased the odds of having post‐COVID cognitive impairment (OR 0.84, 0.83, 0.64, and 0.86 respectively) (all *P<0.0001*). The risk of cognitive diagnosis post‐COVID significantly differed by race with AA males having the highest risk. (all *P<0.0001*).

**Conclusion:**

Our study shows post‐COVID cognitive impairment is common with the top risk factors being AA race, recurrent COVID infections, and cardiovascular disorders. Future studies should investigate the role these risk factors play and their contribution to the mechanisms underlying post‐COVID cognitive impairment.

